# Survival of *Vibrio cholerae* in Nutrient-Poor Environments Is Associated with a Novel “Persister” Phenotype

**DOI:** 10.1371/journal.pone.0045187

**Published:** 2012-09-18

**Authors:** Mohamma Jubair, J. Glenn Morris, Afsar Ali

**Affiliations:** 1 Department of Environmental and Global Health, School of Public Health and Health Profession, University of Florida at Gainesville, Gainesville, Florida; 2 Emerging Pathogens Institute, University of Florida at Gainesville, Gainesville, Florida; Louisiana State University and A & M College, United States of America

## Abstract

In response to antibiotic and/or environmental stress, some species of bacteria shift to a “persister” phenotype. Although toxigenic *Vibrio cholerae*, responsible for the disease cholera, can be found in nutrient-poor aquatic environments in endemic areas, the underlying mechanism(s) by which culturable cells persist in these environmental reservoirs is largely unknown. Here we report that introduction of *V. cholerae* into a nutrient-poor filter sterilized lake water (FSLW) microcosm promoted a shift to what we have defined as a “persister” phenotype (PP) which was culturable for >700 days. Direct transfer of PP of *V. cholerae* from original microcosms to freshly prepared FSLW resulted in the same pattern of persistence seen in the original microcosms. Scanning electron microscopy of cells persisting for over 700 days demonstrated cell morphologies that were very small in size, with a high degree of aggregation associated with flagella emanating from all aspects of the cell. *V. cholerae* PP cells reverted to a typical *V. cholerae* morphology when transferred to nutrient-rich L- broth. Cell-free supernatants obtained from microcosms at 24 hours, 180 days, and 700 days all showed >2-fold increase in CAI-1 signaling molecules, consistent with quorum sensing activity, as has been described for *Pseudomonas aeruginosa* persister cells. Chitin and phosphate promoted cell growth. Our data suggest that nutrient stress can select a *V. cholerae* persister phenotype in environmental reservoirs, with these strains then seeding subsequent cholera epidemics in response to chitin and phosphate availability.

## Introduction

Cholera, an ancient human disease, continues to be a major public health threat worldwide, particularly in countries where sanitary conditions and hygiene are suboptimal [1]. *V. cholerae* strains producing cholera toxin cause the disease cholera, which tends to occur in seasonal epidemics in cholera-endemic regions [2], [3]. Available data suggest that the microorganism survives between epidemics in aquatic reservoirs (including fresh, marine and estuarine waters), with environmental triggers causing seasonal increases in counts, followed by “spill-over” into human populations [1]. This model is complicated, however, by the observation that these environmental reservoirs are often nutrient-poor with extremely low or non-detectable *V. cholerae* cell counts between epidemics or even during epidemics [4], [5]. We still do not have a good understanding of the cellular mechanisms underlying environmental persistence of the microorganism, or of the environmental triggers that stimulate cell growth.

It has been shown that *V. cholerae* enters into a viable but non-culturable state (VBNC) in response to nutrient starvation and cold temperature [6], [7]; however, the resuscitation of VBNC, at least under laboratory conditions, is inconsistent, raising questions about the role of the VBNC state in cholera epidemiology [8], [9]. *V. cholerae* can also switch from a smooth colony type to a “rugose” (wrinkled) colony variant [10], [11]. The rugose variant characteristically produces copious amounts of an exopolysaccharide that confers resistance to chlorine, acid pH, and oxidative and osmotic stresses [12], [13], [14], [15]. Thus, because of the superior survival ability of rugose variant (particularly in stressful environments), compared to their smooth counterpart, we [15] and other investigators [14] have hypothesized that the rugose variant is a survival phenotype of *V. cholerae*. However, that idea is controversial because (i) in our own studies in Bangladesh, the efforts to isolate rugose variants of *V. cholerae* from the bacterium’s aquatic reservoirs have generally not been successful (Ali et al; unpublished observations), and (ii) a previous study [15], using a medium that promotes high-frequency rugose production, found that a majority of the *V. cholerae* strains tested were unable to shift to the rugose state.

For other bacterial pathogens, exposure to adverse growth conditions/antibiotics can promote the emergence of a sub-population of what have been termed “persister” cells, which remain viable for extended periods of time under stress conditions via a variety of mechanisms [16], [17], [18], [19], [20]. In keeping with this persister model, we hypothesize that a subpopulation of *V. cholerae* cells switches to a culturable persister phenotype (PP) or dormant state in response to nutrient starvation upon its release into aquatic environments. In this paper, we present evidence that *V. cholerae* is able to survive in a culturable form for over two years with no added nutrients in a fresh water lake microcosm; that cells in this environment undergo unique morphologic changes, with reversion back to “normal” *V. cholerae* morphology when placed back in nutrient-rich conditions; that they elicit quorum sensing responses, in keeping with prior reports regarding *Pseudomonas aeruginosa* persisters [21], [22]; and that growth is enhanced when strains are exposed to chitin or phosphate.

## Materials and Methods

### Preparation of Nutrient-poor Microcosms

**Figure 1:**
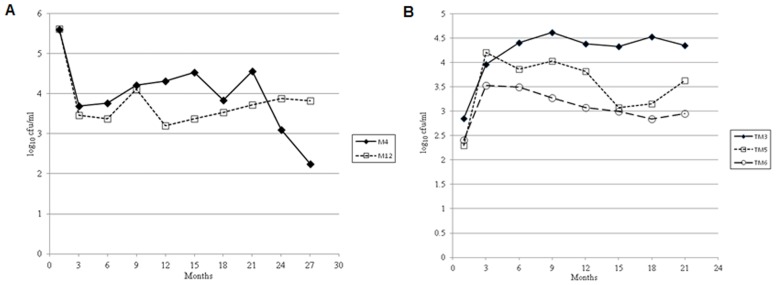
Persistence of epidemic strain of *V. cholerae* N16961 in filter sterilized lake water (FSLW) microcosms stored statically at room temperature. (A) persistence of two microcosms (M4 and M12) for >700 days. (B) persistence of *V. cholerae* in transfer (daughter) microcosms. Transfer microcosm (TM) was prepared by transferring one ml culture from original microcosm (M4, Figure 1A) to fresh FSLW (49-ml) at day 96. The microcosms were stored statically at room temperature. Data represent the persistence of three microcosms in excess of 600 days.

Fresh water used to prepare microcosms was collected from a 30.2-acre natural lake (Wauburg Lake) in Gainesville, Florida. At the time of collection, the pH and the salinity of the water were determined using a portable HACH pH and conductivity meter (model D0175). The pH of collected water ranged from 7.2–8.3. Nutrient composition was determined on two occasions, and was virtually identical both times; Table 1 shows results from one determination, compared to L-broth. The low nutrient content found in our lake water is similar to what has been seen in pond waters obtained from Bangladesh and other regions of the United States [9]. Aliquots (500-ml) of lake water were sterilized using Nalgene 0.22 µm membrane filter units (Nalgene), and microcosms prepared by transferring 50 ml to sterile 250-ml Erlenmeyer flasks. Inocula were prepared by inoculating aliquots (3-ml) of L-broth with a single colony from an overnight plate culture of *V. cholerae* strain N16961. After overnight incubation, cells in L-broth were washed in saline (0.85% NaCl), appropriately diluted, and 100 µl of diluted culture was inoculated into the microcosm flasks. As confirmed by plate counts, initial *V. cholerae* concentrations in the microcosms ranged from 10^4^ to 10^6^ cfu/ml (Figure 1A and Table S1).

**Table 1:** Comparison of the major nutrient components of L-broth and filter sterilized lake water.

Component	L-broth (mg/L)^a^	FS LW (mg/L)^b^
**Total carbohydrate**	1,645	14.3
**Total nitrogen**	1,845	1.55
**Ammonia nitrogen**	820	0.28
**Sodium**	731	8.55
**Chloride**	642	15.0
**Calcium**	2.0	7.48
**Iron**	0.2	1.0
**Potassium**	183	1.43
**Phosphate**	430	0.06
**Magnesium**	120	1.78

a Analysis were performed by Becton and Dickenson (sparks, MD). The data represent the total amount of each component contained in the Bacto Trypton and Yeast extract used to prepare L-broth. The sodium chloride values also include the amount of NaCl used to prepare L-broth.

b The FSLW used in the microcosm assay was analyzed by Advanced Environmental Laboratories, Inc., (Gainesville, Florida, USA).

### Maintenance of Microcosms

Microcosms were maintained at room temperature unless otherwise indicated. The number of persisting culturable *V. cholerae* (cfu/ml) from each microcosm was determined using a standard plate count method; samples for culture were collected daily for the first 8 days after inoculation, and every 15 days thereafter for as long as the microcosm was maintained. Before plating, each microcosm flask was hand-swirled for three minutes to disperse any bacterial cell aggregates that might have been present.

Microcosms in which culturable bacteria were not detected were re-examined for three consecutive days by plating aliquots (100-µl) of undiluted samples on L-agar (10 plates). If no colonies were identified, a final aliquot of 100 µl from each of the microcosms was inoculated into fresh 3-ml L-broth, incubated at 37 and 25°C in both shaking and static growth conditions, and then plated on L-agar with overnight incubation at 37°C. If all plates were negative, we concluded that culturable bacteria were not present in the microcosm. Studies were terminated after 770 days (Figure 1A).

### Transfer (daughter) Microcosms

Twelve “daughter” microcosms, prepared as described above, were inoculated with an aliquot of 1 ml from an original (seed) microcosm (microcosm M4 [Figure 1A and Table S1]) at day 96 of incubation. Samples were collected for plate counts using methods and time intervals as described above for the original microcosms. Studies were terminated at 630 days (Figure 1B and Table S2).

### Scanning Electron Microscopy (SEM)

*V. cholerae* grown either in L-broth or persisting in microcosms at room temperature was fixed with Trumps buffer and deposited onto 0.4 µm polycarbonate membrane filters. The fixed cell samples were washed three times with phosphate buffered saline (PBS; pH 7.24), post fixed with 2% osmium tetroxide, rinsed with deionized water, and dehydrated with ethanol series 25%, 50%, 75%, 95%, 100%. Fixation, rinsing, and dehydration were performed with a Pelco BioWave Pro laboratory microwave (Ted Pella, Redding, CA, USA). Ethanol was removed from the samples by critical point drying (Autosamdri-815, Tousimis Research Corp, Rockville, MD, USA). Dried samples were mounted on carbon adhesive tabs on aluminum specimen mounts and coated with Au/Pd with a sputter coater (DeskV Denton Vacuum, Moorestown, NJ, USA). Micrographs of bacterial cells were acquired with field-emission scanning electron microscope (S-4000, Hitachi High Technologies America, Inc. Schaumburg, IL, USA).

### Quorum Sensing Assay

Quorum sensing assays were performed as described by Miller et al [23]. Briefly, cell-free spent cultures (filtered through a 0.22 µm disposable syringe filter) of *V. cholerae* (ca.10^3^ cfu/ml) persisting in FSLW microcosms at room temperature were used as a source of CAI-1 activity. Supernatants were derived from microcosms persisting for 24 h, 180 days, and 700 days. As a positive control, a single colony of *V. cholerae* N16961 was grown statically overnight in L-broth at 25°C. Control cultures were standardized to ca.10^3 ^cfu/ml by diluting with L-broth, and filter sterilized as described above. Cell-free culture supernatants were tested for the presence of CAI-1 activity by inducing light production in the *V. cholerae* reporter strain MM920 (a kind gift of Bonnie Bassler, Princeton University, PA) containing the cosmid pBB1, which carries the *V. harveyi lux* operon [23]. This reporter strain neither produces CAI-1 nor responds to AI-2. The reporter strain was grown overnight in L-broth media with shaking at 30°C, diluted 1∶10 in fresh L-broth medium, and 70 µl aliquots transferred to an opaque wall 96-well microtiter plate. Thirty µl cell-free supernatant was added to microtiter wells containing 70 µl MM920 culture to obtain a final culture volume of 100 µl. The plates were incubated at 30°C with agitation and light production was measured at 30 min intervals in a BioTek Synergy 2 plate reader (Biotek Instruments, Winooski, VT). Data were reported as peak fold light induction compared to sterile L-broth and lake water controls.

### Supplementation of Major Nutrients to Microcosms

A series of transfer studies were conducted monitoring growth/survival of *V. cholerae* in nutrient-supplemented microcosms. Briefly, an aliquot of 5 µl of *V. cholerae* was directly inoculated into 3-ml of filter sterilized lake water (final concentration of ca. 40–60 cfu/ml) supplemented with different nutrients, as described below. Transfer microcosms were monitored daily for 8 days after inoculation, using inocula from microcosm M4 at day 180 and 700+. Transfer microcosms were supplemented (prior to adding the *V. cholerae* inoculum) with: (i) readily available carbon sources (0.5, 1 and 2% sucrose), (ii) complex carbon sources (alkaline peptone water [APW][a mixture of 1% Peptone and 0.5% Yeast extract, pH 8.6] and chitin [0.05, 0.1 and 0.15%][Sigma-Aldrich, St. Louise, MO]), (iii) nitrogen sources (ammonium bicarbonate [0.5, 1.0 and 1.5 mM] or (iv) phosphate [0.5, 1.0 and 1.5 mM K_2_HPO_4_]. The cultures were incubated statically at room temperature or at 37°C as required and subsequently plated on L-agar. In the initial experiments, we did not find any significant differences in response based on differences in nutrient concentrations. Consequently, we restricted our studies to a single final concentration of representative compounds: (i) sucrose (1%); (ii) 1X APW; (iii) chitin (.05%); (iv) ammonium bicarbonate (1.0 mM); and (v) phosphate (1.0 mM).

## Results

### Persistence of Culturable *V. cholerae* in Lake Water Microcosms as “Persister Phenotype”

We prepared fifteen original independent microcosms. Eight of the 15 microcosms were culture-negative within 9 days of inoculation; 5 become culture negative within 120 days (Table S1), while in two instances culturable *V. cholerae* were detectable for over 700 days (Figure 1A). To explore the persister phenotype’s response and adaptation to fresh FSLW, 1 ml inocula were transferred on day 96 from microcosm M4 to twelve fresh 50 ml FSLW microcosms. *V. cholerae* in these transfer (“daughter”) microcosms showed an initial increase in counts, from ca. 10^2^ to ca. 10^4^ cfu/ml (Figure 1B and Figure S2 B) suggesting that starved *V. cholerae* in microcosms can response to miniscule amounts of nutrients contained in FSLW. Subsequent patterns of survival in these daughter microcosms were similar to those seen with the initial microcosms, with 2 of the 12 showing death of the microcosm within 8 days, 7 of the 12 exhibiting death between 9 and 220 days (Table S2), and 3 having culturable *V. cholerae* for periods in excess of 600 days (Figure 1B). Microorganisms recovered from all microcosms were confirmed as *V. cholerae*, based on serologic and genetic analysis, including screening for *toxR* and *ompW* (*V. cholerae* species-specific genes) and *tcpA* and *ctxB* (*V. cholerae* virulence genes).

### Morphology of *V. cholerae* Persisting in Microcosms

**Figure 2:**
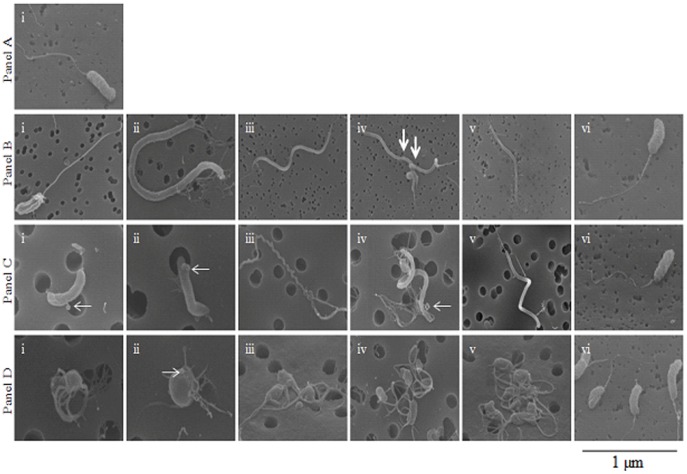
Scanning electron micrographs of *V. cholerae* strain N16961. Panel A: Scanning electron microscopy (SEM) image of *V. cholerae* grown statically overnight at room temperature in L-broth. Panel B: Images obtained with SEM from *V. cholerae* grown overnight statically at room temperature in FSLW microcosm. Images i to v exhibit diverse *V. cholerae* morphologies. Image vi obtained after an aliquot of 100 µl of the 24 h old microcosm was transferred to L-broth and incubated statically overnight at room temperature before SEM was performed. Panel C: Images obtained with SEM from *V. cholerae* persisting statically at room temperature in microcosm for 180 days. Images i through v exhibit different *V. cholerae* morphologies. Image vi obtained after an aliquot of 100 µl of the 180 days old microcosm was transferred to L-broth and incubated overnight at room temperature before SEM was performed. Panel D: Images obtained with SEM from *V. cholerae* persisting statically at room temperature in microcosm for 700 days. Images i through v exhibit different *V. cholerae* morphologies. Image vi obtained after an aliquot of 100 µl of the 700-day old microcosm was transferred to L-broth and incubated overnight at room temperature before SEM was performed. (scale bar, 1 µm; thick arrows indicate evidence of cell division; thin arrows indicate bud and OMVs formation).

As *V. cholerae* persisted for over 700 days in microcosms, we examined the cellular morphology of the bacterium at multiple survival points. When examined by scanning and transmission electron microscopy, material from microcosms showed evidence of morphologic changes in cells within 24 hours of introduction into the nutrient-poor environment: this includes what appeared to be stressed *V. cholerae* cells with a single polar flagellum, and elongated and helical cells with predominantly bipolar flagella with evidence of cell division in elongated cells (Figure 2, panel B). Material from microcosms that retained culturability at 180 days showed (i) no evidence of cell division, (ii) formation of predominantly helical and spiral cells with a very limited number of coccoid cells, including cells producing and disseminating what appeared to be numerous outer membrane vesicles and buds [24], (iii) a high degree of pilliation, (iv) replacement of a singular polar flagellum with bipolar and peritrichous flagella, and (v) non-flagellated curved and rod shaped cells (Figure 2, panel C). At 700 days, the majority of cells were very small in size, with a high degree of aggregation associated with pleotrophic flagella emanating virtually from all sides of the cells (Figure 2, panel D). Direct transfer into L-broth of microcosm material (100 µl) from each of these time points resulted in reversion to a cell morphology (Figure 2, frame at far right of panels B, C, and D) indistinguishable from that of the original inoculum (Figure 2, panel A). Material from “dead” microcosms, from which *V. cholerae* could not be isolated, showed only cellular debris, with no evidence of intact cells, including no evidence of the small, coccoid cells that have been associated with the viable but non-culturable phenotype [6]. When material from the 700+ day microcosms was directly plated on L-agar, *V. cholerae* colonies showed a reduction of 75% in colony size, as compared with colonies recovered from samples collected from the original inoculum and from microcosms through about day 300 after inoculation. These small colonies reverted to normal colony size when passed a second time on L-agar (Figure S1).

### Quorum Sensing

**Figure 3:**
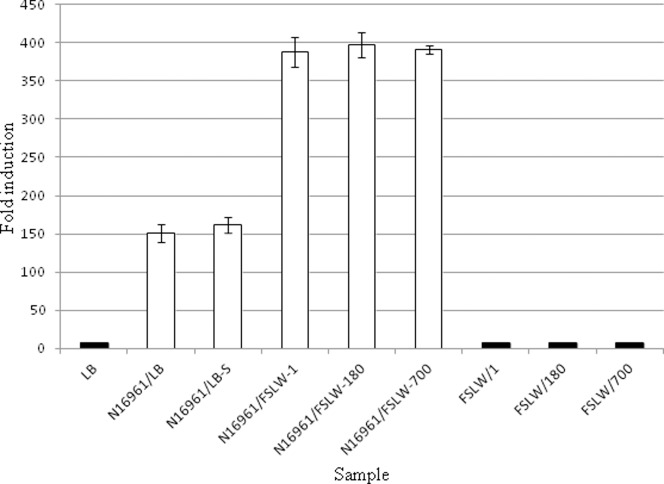
Effect of quorum sensing mechanism(s) on the persistence of *V. cholerae* in FSLW microcosm. Measurement of CAI-1 activity in *V. cholerae* grown either overnight in L-broth at 37°C with a shaking speed of 250 rpm or in *V. cholerae* persisting in FSLW statically at room temperature for 1 day (24 h), 180 days and 700 days. Data represent the average results obtained from 6 independent experiments. Cell-free spent media were used as sources of CAI-1 molecules that induced light production in *V. cholerae* strain MM920 as described previously [23]. LB, sterile L-broth without reporter strain, MM920; N16961/LB, overnight grown *V. cholerae* in L-broth was diluted appropriately (to obtain ca. 10^3^ cfu/ml) in L-broth and the diluted sample was filter sterilized and used, N16961/LB-S; cell-free spent medium of *V. cholerae* grown overnight in L-broth was diluted in L-broth appropriately and used; N16961/FSLW-1, *V. cholerae* (ca. 10^3^ cfu/ml) was grown overnight statically at room temperature in FSLW microcosm; N16961/FSLW-180, cell-free supernatant was obtained from *V. cholerae* persisting for 180 days in FSLW microcosm; N16961/FSLW-700, cell-free supernatant was obtained from *V. cholerae* persisting for 700 days in FSLW microcosm; FSLW/1, 100 µl FSLW without reporter strain; FSLW/180, 100 µl sterile spent media obtained from 180 days old microcosm without reporter strain inoculation; FSLW/700, 100 µl sterile spent media obtained from 700 days old microcosm without reporter strain inoculation. Bar indicates standard error.

Formation of persister cells in *Pseudomonas aeruginosa* has been linked with a quorum sensing mechanism [21], [22]. To determine if *V. cholerae* persister cells produce quorum sensing signals in adapting to nutrient stressed microcosm condition, we measured CAI-1 signaling molecules in cell-free spent microcosm materials using *V. cholerae* reporter strain MM920, as previously described [23]. Cell-free supernatants obtained from microcosms at 24 hours, 180 days, and 700 days all showed >2-fold increase in activity as compared with sterile spent culture media from L-broth stationary cultures (Figure 3).

### Effect of Nutrients on the Growth of *V. cholerae* Persisting in Microcosms

**Figure 4:**
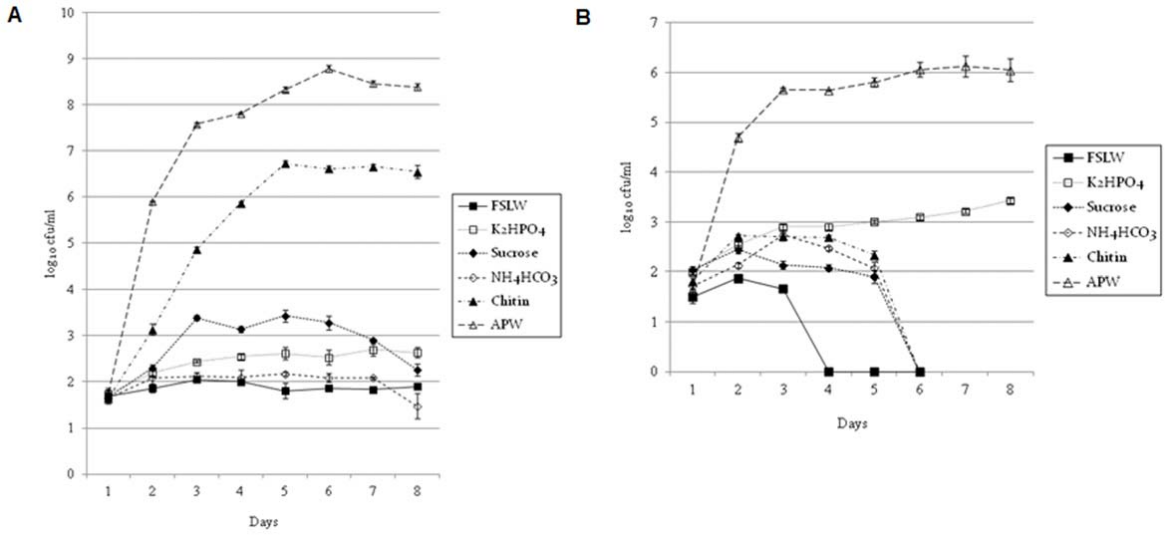
Effect of fresh FSLW supplemented with nutrients on the growth and persistence of *V. cholerae* surviving in microcosms incubated statically at room temperature for 180 and 700 days. Data represent the average result obtained from six independent microcosms: (A) Five µl inocula (ca. 40–60 cfu/ml) from microcosms persisting for 180 days were directly transferred to 3 ml fresh FSLW supplemented with indicated nutrients. Counts (cfu/ml) of culturable *V. cholerae* were determined for 8 consecutive days using standard plate count, and (B) indicated nutrients were added to the fresh FSLW prior to transfer of 5 µl inocula (ca. 40–60 cfu/ml) from microcosm persisting for 700 days. Counts (cfu/ml) of culturable *V. cholerae* were determined for 8 consecutive days using standard plate count. Bar indicates standard error.

When either chitin or potassium phosphate was added to the FSLW prior to the inoculation of materials from the original microcosm (inocula taken from both 180 and 700+ days), *V. cholerae* showed an increase in counts (Figure 4A and 4B, respectively). Although both chitin and phosphate promoted the growth of nutritionally stressed *V. cholerae* in microcosms, there were differences in responses when inocula from 180 and 700+ days were compared. Bacteria from 180 days (still showing a typical large colony morphology; Figure S1) showed the most striking response to chitin (Figure 4A). In contrast, bacteria from 700+ day microcosms (which had a small colony morphology; Figure S1) exhibited much more prominent response to phosphate (Figures 4B and figures S3 A, B, C). Interestingly, a more readily available carbon source (sucrose) was able to only poorly promote the growth of *V. cholera*e. In contrast, complex organic material present in APW promoted the robust growth of the organism at all experimental conditions. Our results suggest that once *V. cholerae* senses a nutrient-poor environment, it immediately turns off pathways needed to metabolize inorganic simple carbohydrate, while turning on genetic mechanisms required to metabolize complex carbohydrate. We, however, cannot rule out the possibility that, in addition to complex carbohydrate, proteins, minerals, and vitamins in APW contributed to the robust growth of the bacterium.

We were unable to induce culturable *V. cholerae* cells from apparently dead microcosms when the microcosms were supplemented with nutrients used in our experiments. Our results corroborate the earlier report that once culturable *V. cholerae* becomes non-detectable in microcosms, it cannot be resuscitated into a culturable form using methods employed in our study [25].

To determine the effect of temperature on the persistence of *V. cholerae* in microcosms, we incubated microcosms at 37°C. Compared to microcosms incubated at 25°C, incubation of microcosms at 37°C without nutrient supplementation resulted in a much more rapid die-off of culturable *V. cholerae* (Figure S2 A); however, an increase in counts was seen with the addition of either chitin or phosphate (Figures S3 A, B, C). Our results suggest that while temperature alone may not induce the growth of *V. cholerae* in nutrient poor conditions, a combination of nutrients (chitin and/or phosphate), complex carbohydrates, and temperature may play a major role in the increase of the microorganism.

## Discussion

The key question asked in this study was: Does *V. cholerae* retain a culturable phenotype in response to nutrient starvation, and if so what morphological and physiological changes occur in association with this phenotype? As monitoring of *V. cholerae* survival in the natural environment is difficult (as reservoirs may differ widely in nutrient content, temperature, and a range of other variables), we used a lake water microcosm model that permitted careful monitoring of survival across time, in a way that would not be possible outside of the laboratory. At the same time, it may also limit the range of responses: under different environmental conditions, *V. cholerae* may respond in different ways (including elicitation of the viable but non-culturable phenotype, which we did not observe). Consistent with our hypothesis, we demonstrated that, in response to nutrient limitation, *V. cholerae* promotes and selects a phenotype that fits the classic definition of the persister model seen with other bacteria [17], [18]. The cells described here survived for over 700 days in lake water microcosms without loss of culturability. In contrast to our findings, in a microcosm study reported by other investigators, culturable *V. cholerae* was detected for 85 days, with viable but non-culturable (VBNC) cells identified for an additional 495 days, after which the VBNC cells became undetectable [25]. It remains to be seen how these different mechanisms contribute to the seasonal patterns of epidemic cholera seen in endemic regions.

In keeping with a “persister” conceptual framework [17], [18], which implies a stochastic origin, cells transferred from persisting microcosms into new nutrient-poor FSLW showed patterns of survival that mirrored those seen among the initial microcosms. We did see evidence that persisting cells underwent morphologic changes, beginning as early as 24 hours after introduction into a nutrient-poor environment, and progressing across time to novel morphologic types characterized by small cell size, aggregation, and pleotrophic flagella by 700 days. However, when persisting cells were placed back into rich media, they regained a “normal” microscopic appearance. Similarly, microcosm material directly plated on L-agar had a small colony morphology, which reverted to a normal morphology when re-plated. The genetic basis and drivers for these morphologic changes remains to be determined: among other possibilities, it may reflect action of a two-component genetic regulatory system, genetic switching, epigenetic mechanisms, or even a single nucleotide mutation. Indeed, a study using an advanced genetic screening mechanism and subsequent knock out mutation demonstrated that global regulators, including DksA, DnaKJ, HupAB, and IhfAB were involved in the persister formation in *Escherichia coli* [26].

Our data suggest that quorum sensing plays a role in this process, as has been described for *Pseudomonas aeruginosa* strains having a persister phenotype [21]. Although we have seen a significant increase (2-fold) in quorum sensing molecules in *V. cholerae* persisting in microcosms compared to that of in L-broth grown cells, we observed no difference in signaling molecules when microcosm materials were examined at 1, 180, and 700 days post inoculation. This may reflect continuous production of controlled amounts of quorum sensing molecules or alternatively, the CAI-1 molecules are highly stable in under conditions present in the microcosm, reducing the rate of decline once produced by *V. cholerae* in the early phase of persistence. Interestingly, quorum sensing mechanisms/signaling molecules increased the persistence of *Pseudomonas aeruginosa* [27], [22]. Again, further molecular studies will be needed to confirm and define the mechanisms involved, and the role of quorum sensing in the persister state.

In environmental studies conducted by our group and others, there is clear evidence that seasonal cholera epidemics are preceded by increases in *V. cholerae* in environmental reservoirs [28]. This appears to be mediated, at least in some areas, by changes in water temperature [4]. However, we found that elevated temperatures (37°C) reduced survival in our microcosm model, potentially due to temperature-dependent increases in metabolism and stress in this nutrient-poor environment [29]. We did find that growth of persisting cells was differentially facilitated by chitin (a complex carbon source), phosphate, and the presence of complex carbohydrates. Chitin is well recognized as a key trigger for natural competency and biofilm formation in *V. cholerae* [30]. The link with chitin would be in keeping with prior work suggesting that occurrence of seasonal epidemics is triggered by blooms of phytoplankton, followed by zooplankton such as copepods, events which would increase exposure to chitin [4]. Similarly, environmental phosphate concentrations tend to be driven by variables such as rainfall (linked with agricultural runoff), and/or dry season conditions that decrease water levels in ponds and other potential reservoirs [31], [32]; both events are variables that have been linked with initiation of epidemics [31]. In addition to phosphate, heavy rainfall and floods may also enrich water with complex carbohydrates that could promote the growth of starved culturable *V. cholerae* leading to the initiation of cholera epidemics.

## Supporting Information

Figure S1 **Photographs of** ***V. cholerae*** **N16961 colonies on L- agar plates incubated overnight at 37°C.** (**A)** *V. cholerae* colonies on L-agar (colony diameter ranges from 2 to 2.5 mm), (**B)** *V. cholerae* colonies on L-agar after persisting in 180 days in microcosm (colony diameter ranges from 2 to −2.5 mm), **(C)** *V. cholerae* colonies growing on L-agar after persisting in 700 days in microcosm (colony diameter ranges from 0.5 to 0.6 mm), (**D)** an aliquot (100 µL) of 700 days microcosm was transferred into 3 ml of L-broth and incubated at room temperature. Subsequently the culture was plated on L-agar (colony diameter ranges from 2 to 2.5 mm).
(TIF)

Figure S2 **Persistence of** ***V. cholerae*** **in the FSLW either in fresh FSLW or in FSLW supplemented with nutrients.** The microcosms were incubated at either room temperature or at 37°C: (**A**) Comparison of the persistence of *V. cholerae* in FSLW microcosms incubated either at room temperature or at 37°C. Counts (cfu/ml) of culturable *V. cholerae* were taken each day for 8 consecutive days using standard plate count. The results represent the average viable counts obtained from eight independent microcosms, (**B**) one ml of microcosm material was transferred from an original microcosms (M4) to fresh 49-ml FSLW. The microcosms were incubated statically at room temperature. Counts (cfu/ml) of culturable *V. cholerae* were taken each day for 8 consecutive days using standard plate count. The results represent the average viable counts obtained from six independent microcosms.
(TIF)

Figure S3 **Effect of major nutrients on the growth and persistence of** ***V. cholerae*** **strain N16961 in the FSLW microcosms incubated at 37°C for 8 days.** Data represent the average result obtained from three independent microcosms except for control microcosm (without added nutrients) where data represent the average result of eight independent microcosms: (**A**) indicated nutrients were added to the fresh FSLW before adding the inoculums (ca. 10^5^–10^6^ cfu/ml), **(B**) indicated nutrients were added to the fresh FSLW before direct transfer of 5 µl inoculum (ca. 40–60 cfu/ml) from 180 days old microcosm. As controls, FSLW containing no added nutrients were also inoculated with 5 µl inoculums directly from 180 days old microcosm, (**C**) indicated nutrients were added to the fresh FSLW before direct transfer of 5 µl inoculum (ca. 40–60 cfu/ml) from 700 days old microcosm. As controls, FSLW containing no added nutrients were also inoculated with 5 µl inoculums directly from 700 days old microcosm.
(TIF)

Table S1 **Lake water microcosms.** Persistence of *V. cholerae* strain N16961 in filter sterilized lake water microcosm (original microcosms)
(DOCX)

Table S2 **Transfer microcosms.** Persistence of *V. cholerae* strain N16961 in transfer (daughter) microcosm (TM).
(DOCX)
