# Correction: Survival of *Vibrio cholerae* in Nutrient-Poor Environments Is Associated with a Novel “Persister” Phenotype

**DOI:** 10.1371/annotation/cb679494-d0cd-4658-a0a1-ab548c6e7139

**Published:** 2012-12-05

**Authors:** Mohamma Jubair, J. Glenn Morris, Afsar Ali

The first author's name was spelled incorrectly. The correct name is: Mohammad Jubair.

