# Supplementary figures and images for: Survival of Vibrio cholerae in Nutrient-Poor Environments Is Associated with a Novel “Persister” Phenotype
Source: PLoS One. 2012 Sep 18;7(9):e45187. doi: 10.1371/journal.pone.0045187 (PMC3445476; doi:10.1371/journal.pone.0045187)

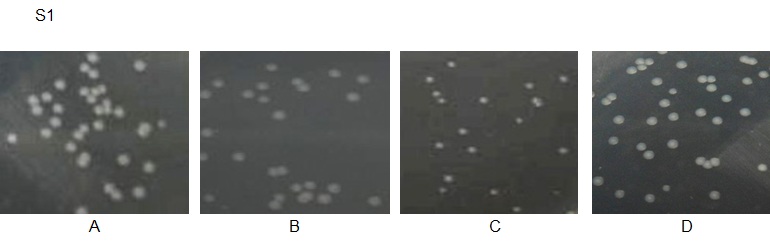

Supplement: Figure S1 — Photographs of V. cholerae N16961 colonies on L- agar plates incubated overnight at 37°C. (A) V. cholerae colonies on L-agar (colony diameter ranges from 2 to 2.5 mm), (B) V. cholerae colonies on L-agar after persisting in 180 days in microcosm (colony diameter ranges from 2 to −2.5 mm), (C) V. cholerae colonies growing on L-agar after persisting in 700 days in microcosm (colony diameter ranges from 0.5 to 0.6 mm), (D) an aliquot (100 µL) of 700 days microcosm was transferred into 3 ml of L-broth and incubated at room temperature. Subsequently the culture was plated on L-agar (colony diameter ranges from 2 to 2.5 mm). (TIF) [file pone.0045187.s001.tif]

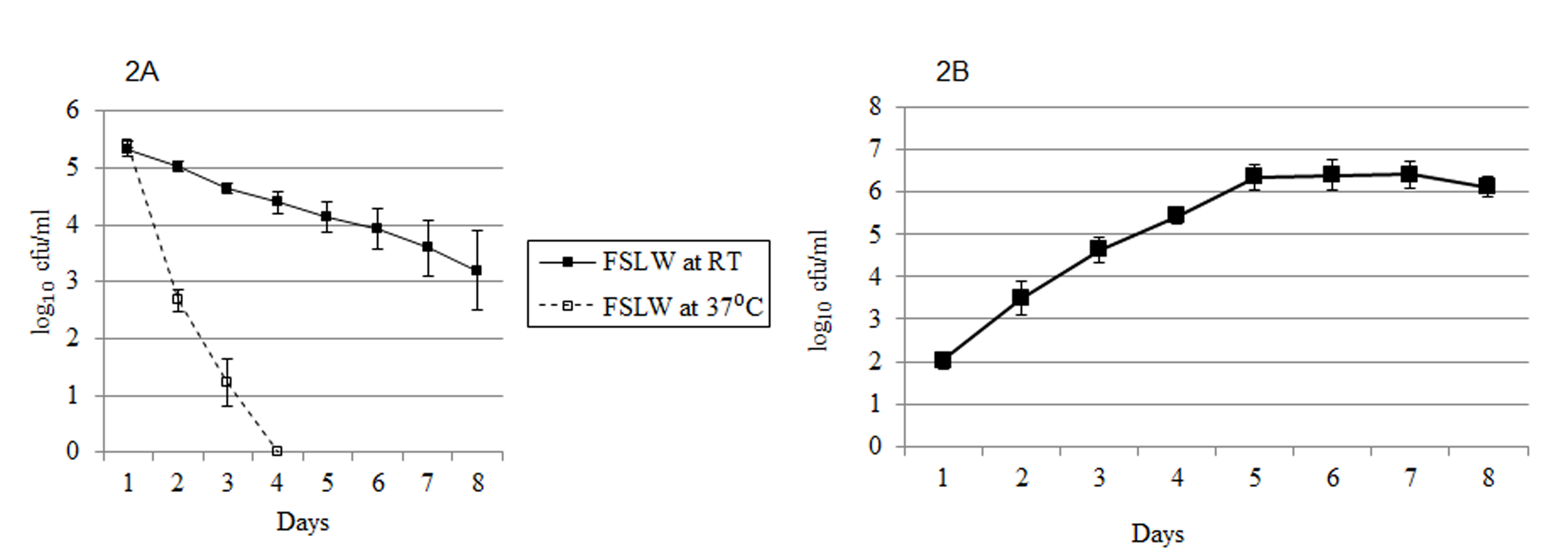

Supplement: Figure S2 — Persistence of V. cholerae in the FSLW either in fresh FSLW or in FSLW supplemented with nutrients. The microcosms were incubated at either room temperature or at 37°C: (A) Comparison of the persistence of V. cholerae in FSLW microcosms incubated either at room temperature or at 37°C. Counts (cfu/ml) of culturable V. cholerae were taken each day for 8 consecutive days using standard plate count. The results represent the average viable counts obtained from eight independent microcosms, (B) one ml of microcosm material was transferred from an original microcosms (M4) to fresh 49-ml FSLW. The microcosms were incubated statically at room temperature. Counts (cfu/ml) of culturable V. cholerae were taken each day for 8 consecutive days using standard plate count. The results represent the average viable counts obtained from six independent microcosms. (TIF) [file pone.0045187.s002.tif]

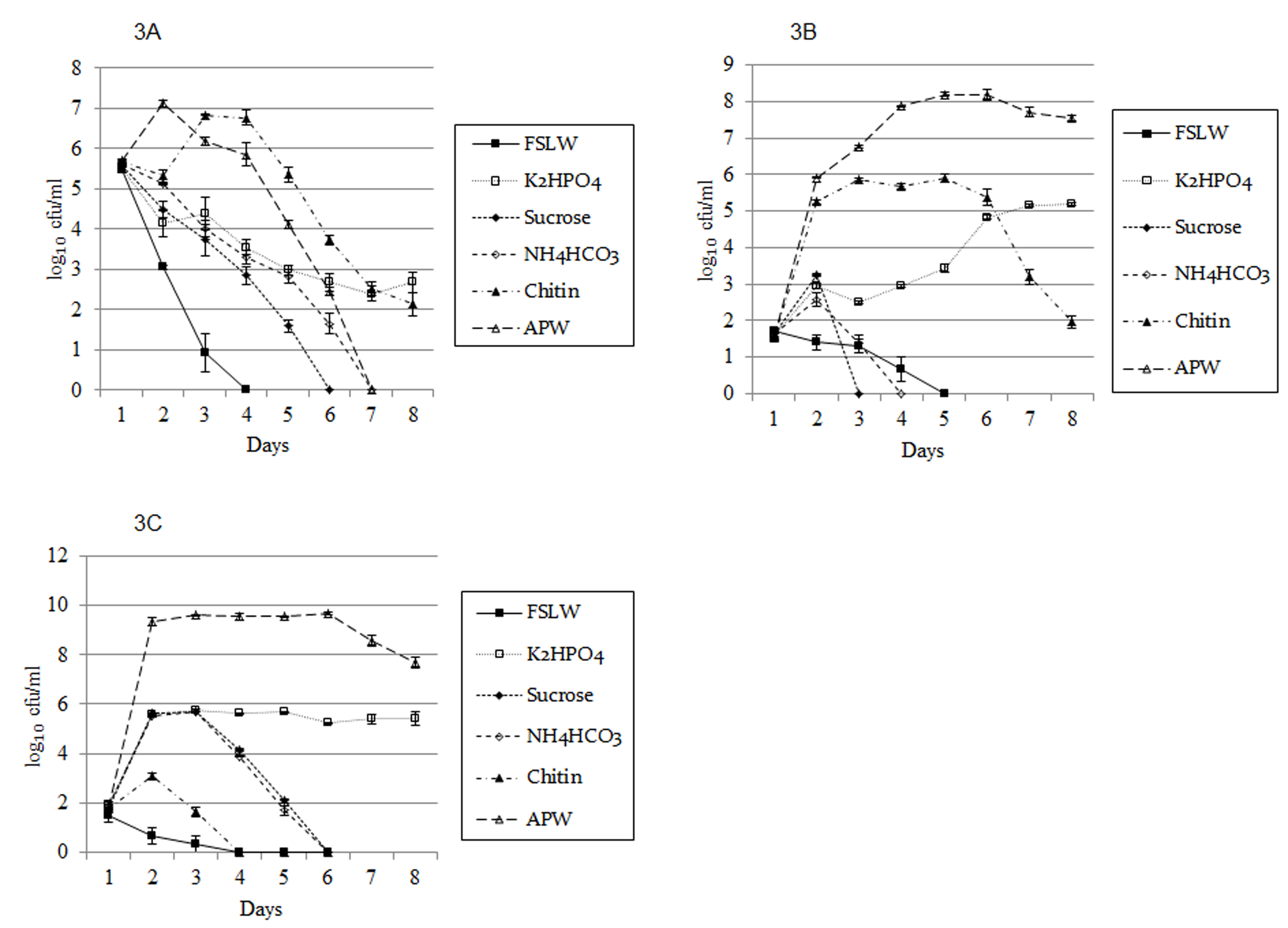

Supplement: Figure S3 — Effect of major nutrients on the growth and persistence of V. cholerae strain N16961 in the FSLW microcosms incubated at 37°C for 8 days. Data represent the average result obtained from three independent microcosms except for control microcosm (without added nutrients) where data represent the average result of eight independent microcosms: (A) indicated nutrients were added to the fresh FSLW before adding the inoculums (ca. 105–106 cfu/ml), (B) indicated nutrients were added to the fresh FSLW before direct transfer of 5 µl inoculum (ca. 40–60 cfu/ml) from 180 days old microcosm. As controls, FSLW containing no added nutrients were also inoculated with 5 µl inoculums directly from 180 days old microcosm, (C) indicated nutrients were added to the fresh FSLW before direct transfer of 5 µl inoculum (ca. 40–60 cfu/ml) from 700 days old microcosm. As controls, FSLW containing no added nutrients were also inoculated with 5 µl inoculums directly from 700 days old microcosm. (TIF) [file pone.0045187.s003.tif]
