# Supplementary material for: Survival of Vibrio cholerae in Nutrient-Poor Environments Is Associated with a Novel “Persister” Phenotype
Source: PLoS One. 2012 Sep 18;7(9):e45187. doi: 10.1371/journal.pone.0045187 (PMC3445476; doi:10.1371/journal.pone.0045187)
Supplement: Table S1 — Lake water microcosms. Persistence of V. cholerae strain N16961 in filter sterilized lake water microcosm (original microcosms) (DOCX) [file pone.0045187.s004.docx]

Table S1

| Microcosm (M) | log_10_cfu/ml^a^ | Persistence (in days)^b^ |
| --- | --- | --- |
| M1 | 5.14 | 9 |
| M2 | 4.83 | 8 |
| M3 | 5.24 | 8 |
| M4 | 5.6 | 770 |
| M5 | 4.6 | 4 |
| M6 | 4.48 | 3 |
| M7 | 4.34 | 4 |
| M8 | 5.57 | 96 |
| M9 | 5.05 | 120 |
| M10 | 5.23 | 120 |
| M11 | 4.96 | 120 |
| M12 | 5.62 | 770 |
| M13 | 5.15 | 6 |
| M14 | 4.85 | 5 |
| M15 | 5.2 | 22 |

^a^Number of culturable *V. cholerae* colony from each individual microcosm was determined using the standard plate count immediately after adding the inoculum to the microcosm.

^b^Days after which the culturable *V. cholerae* in microcosm became non-detectable.
