# Supplementary material for: Survival of Vibrio cholerae in Nutrient-Poor Environments Is Associated with a Novel “Persister” Phenotype
Source: PLoS One. 2012 Sep 18;7(9):e45187. doi: 10.1371/journal.pone.0045187 (PMC3445476; doi:10.1371/journal.pone.0045187)
Supplement: Table S2 — Transfer microcosms. Persistence of V. cholerae strain N16961 in transfer (daughter) microcosm (TM). (DOCX) [file pone.0045187.s005.docx]

Table S2

| Transfer microcosm (TM) | log_10_cfu/ml^a^ | Persistence (in days)^b^ |
| --- | --- | --- |
| TM1 | 2.64 | 29 |
| TM2 | 2.61 | 3 |
| TM3 | 2.36 | 630 |
| TM4 | 2.34 | 111 |
| TM5 | 2.3 | 630 |
| TM6 | 2.41 | 630 |
| TM7 | 1.3 | 98 |
| TM8 | 1 | 7 |
| TM9 | 2.08 | 71 |
| TM10 | 2.11 | 33 |
| TM11 | 1.3 | 33 |
| TM12 | 1 | 220 |

^a^ Number of culturable *V. cholerae* colony from each individual microcosm was determined using the standard plate count immediately after adding the inoculum from original microcosm (M4) to freshly prepared FSLW.

^b^Days after which the culturable *V. cholerae* in microcosm became non-detectable.
